# The Homœopathic Treatment of Diphtheria

**Published:** 1881-12

**Authors:** 


### BOOK NOTICES, REVIEWS, ETC.

THE HOMŒOPATHIC TREATMENT OF DIPHTHERIA. By De Forest Hunt, M. D., pp. 94, Eaton, Lyon & Co.: Grand Rapids, Michigan, 1880.

The author, in the preface, says: "In this manual, I have endeavored to furnish an outline of pure homœopathic treatment; and every remedy mentioned has been carefully selected according to its characteristic indications." Under "Treatment" we read: "Our distinctiveness as homœopathic physicians, consists in our therapeutic law of similars; and, by the application of this law to diphtheria, we discover, in the homœopathic materia medica, the means of treating this disease." No gargles, washes, or such like allopathic expedients are mentioned, as Dr. Hunt knows none of them are needed.

Very clear and brief articles are given on diagnosis, prognosis (showing how more successful is homœopathy, than allopathic expedients), and the symptomatology of some twenty remedies.

This is a good manual, but it has the same fault as those of Gregg & McNeil—*no repertory.*

